# Influencing Factors of PM_2.5_ Pollution: Disaster Points of Meteorological Factors

**DOI:** 10.3390/ijerph16203891

**Published:** 2019-10-14

**Authors:** Ruiling Sun, Yi Zhou, Jie Wu, Zaiwu Gong

**Affiliations:** 1 School of Applied Meteorology, Nanjing University of Information Science & Technology, Nanjing 210044, China; 2 Nanjing Research Institute of Ecological and Environmental Protection, Nanjing 210013, China; 3 Jiangsu Institute of Quality & Standardization, Nanjing 210029, China; 4 School of Management Science and Engineering, Nanjing University of Information Science & Technology, Nanjing 210044, China

**Keywords:** disaster point, PM_2.5_, meteorological factors, human activities, stochastic DEA

## Abstract

A chance constrained stochastic Data Envelopment Analysis (DEA) was developed for investigating the relations between PM_2.5_ pollution days and meteorological factors and human activities, incorporating with an empirical study for 13 cities in Jiangsu Province (China) to illustrate the model. This approach not only admits random input and output environment, but also allows the evaluation unit to exceed the front edge under the given probability constraint. Moreover, observing the change in outcome variables when a group of explanatory variables are deleted provides an additional strategic technique to measure the effect of the remaining explanatory variables. It is found that: (1) For 2013–2016, the influencing factors of PM_2.5_ pollution days included wind speed, no precipitation day, relative humidity, population density, construction area, transportation, coal consumption and green coverage rate. In 2016, the number of cities whose PM_2.5_ pollution days was affected by construction was decreased by three from 2015 but increased according to transportation and energy utilization. (2) The PM_2.5_ pollution days in southern and central Jiangsu Province were primarily affected by the combined effect of the meteorological factors and social progress, while the northern Jiangsu Province was largely impacted by the social progress. In 2013–2016, at different risk levels, 60% inland cities were of valid stochastic efficiency, while 33% coastal cities were of valid stochastic efficiency. (3) The chance constrained stochastic DEA, which incorporates the data distribution characteristics of meteorological factors and human activities, is valuable for exploring the essential features of data in investigating the influencing factors of PM_2.5_.

## 1. Introduction

PM_2.5_ refers to particles in the atmosphere of less than or equal to 2.5 microns in diameter. Every autumn and winter, the Ministry of Ecology and Environment (MEEC) of China releases regularly information regarding heavy air pollution conditions, especially fine particulate matter [1]. According to these reports, heavy pollution weathers have significantly decreased in recent years in terms of the frequency and duration, indicating that the preventive and control strategies boost some substantial progress.

On the other hand, according to the Chinese Environmental Status Bulletin, from 2013 to 2016, the annual average concentration of PM_2.5_ in China is 57.75 μg/m^3^ (8.47 μg/m^3^ in the USA, 10.00 recommended by the World Health Organization (WHO) [2]). Long-term exposure to PM_2.5_ pollution has a significant impact on the health of human beings, especially infants and juveniles [3]. Therefore, it is necessary to study the influencing factors of PM_2.5_ and effectively control PM_2.5_ pollution.

In recent years, due to the impacts of PM_2.5_ pollution on human health [4,5,6,7], research on how to control and reduce PM_2.5_ pollution has gradually increased. Some studies have shown that since PM_2.5_ concentration is greatly affected by meteorological conditions [8], climate change (including temperature, precipitation, etc.) has a certain impact on PM air quality [9,10]. However, meteorological conditions are uncontrollable factors. In order to take effective strategies to control PM_2.5_ pollution, it is first necessary to identify and quantify the main sources of fine particulate matter. For example, in order to control PM_2.5_ pollution in the Los Angeles basin, the researchers utilized Positive Matrix Factorization (PMF) to quantify sources of ambient PM_2.5_ in central Los Angeles and Rubidoux, concluded that vehicular emissions (including gasoline and diesel vehicles) were the second major contributor to PM_2.5_, following secondary aerosols [11]. For regional PM_2.5_ pollution problems, some studies have tested and compared PM_2.5_ concentration in cities and surrounding areas and investigated the impact of urbanization on PM_2.5_ concentration in cities in various counties in China [12]. Some studies have also concluded that if the extensive development model is adhered to, economic growth, industrialization and urbanization will inevitably lead to increased PM_2.5_ emissions in the long term [13]. At the same time, some studies have also concluded that fine particulate matter (PM_2.5_) is also influenced by socioeconomic development, in addition to pollutant emissions and meteorological conditions [14].

### 1.1. Meteorological Factors

Although different countries have different PM_2.5_ pollution levels and meteorological conditions, the judgment that PM_2.5_ concentration depends on local meteorological conditions is consistent for different countries. Tai et al. [9] studied the correlations between fine particulate matter (PM_2.5_) and meteorological variables in the United States and obtained the implications for the sensitivity of PM_2.5_ to climate change. Wang and Ogawa [8] analyzed the effects of meteorological conditions on PM_2.5_ concentrations in Nagasaki, Japan. Gao et al. [10] studied the response of winter fine particulate matter concentrations to emission and meteorological changes in North China. In general, the effect of meteorological factors on PM_2.5_ concentrations has become a hot topic in recent years. Some studies have examined the effects of a single [15], multiple [16,17] or various meteorological factors on PM_2.5_ by using the statistical analysis (SA), multifractal detrended cross-correlation analysis (MF-DCCA), and so on. For instance, using the conditional bivariate probability function analysis, coherence wavelet transform and Hybrid Single-Particle Lagrangian Integrated Trajectory (HYSPLIT) back trajectory model, Sumesh et al. [18] found that the concentration of particulate matters was negatively correlated with wind speed, precipitation and relative humidity, while there was a positive relation between high PM_2.5_ concentrations and low wind speed indicating the presence of local pollutants. Based on the correlation analysis method, Galindo et al. [19] found that the daily and seasonal changes in particulate matter concentration were related to the seasonal changes of meteorological conditions: In the winter, there was a good negative correlation with the wind speed. The coarse particulate matters were related to temperature and solar radiation. In addition, the influence of meteorological factors on severe haze weather formation was also studied. Zhang et al. [20] and Mu and Zhang [21] found that the severe haze event in China in January 2013 was closely related to meteorological factors (wind, temperature, humid air, etc.). Dimitriou and Kassomenos [22] studied the relationship between ground-based measurements of particle concentrations (PM_10_ and PM_2.5_) and meteorological parameters (air temperature, wind speed, relative humidity and visibility). Liang and Tang [23] found that the weather in the southern part of Northern China was conducive to the occurrence of haze weather. About seasonal or regional characteristics PM_2.5_, some have focused on the influence of seasonal or regional meteorological factors on the regional PM_2.5_ concentration, and found that regional atmospheric conditions, seasonal and geographical factors have considerable impacts on the PM_2.5_ [24,25,26,27].

Meteorological conditions affect PM_2.5_ concentration, which in turn affects the entire polluted weather process. This weather pollution process may be one day, several days or ten days. It is necessary to study the relationship between meteorological conditions and PM_2.5_ pollution days (according to China’s ambient air quality standards (GB3095-2012), when the 24 h average concentration of PM_2.5_ exceeds 75 μg/m^3^, it is a PM_2.5_ pollution day), but this kind of research is rare or essentially absent. This study assumed that the PM_2.5_ pollution days in a region is related to meteorological conditions, and based on this assumption, we studied the relationship between them.

### 1.2. Human Activities

Emissions from human activities are the dominant factors of PM_2.5_ pollution [28], but human activities in different regions have different effects on PM_2.5_ [29]. First, the contribution of single human activity to PM_2.5_ varies greatly. For instance, some have studied the relationship between vehicle emissions and PM_2.5_ [30,31]. In addition, Guo et al. [32] found that the expansion of logistic services had the greatest impact on air pollution, but Gross Domestic Product (GDP) and urban population growth were only weakly correlated with air pollution. Gautam et al. [33] found that the higher sulfate, nitrate and ammonium (SNA) and organic matter (OM) content in PM_2.5_ (contributed over 40 and 35%) results from heavy traffic, vehicle emissions and burning of solid fuel in most parts of China. Negral et al. [34] found that PM_10_ and PM_2.5_ in the atmosphere were derived from crustal materials and traffic emissions. Xia et al. [35] has shown that the spatial form of urban construction land influences PM_2.5_ diffusion through wind speed, resulting in uneven distribution of PM_2.5_.

Second, complex human activities, such as social and economic development, have a significant impact on PM_2.5_ in different regions [36,37,38,39]. Some scholars have studied the contribution of different human activities to PM_2.5_ from the perspective of source analysis. For instance, Jin et al. [40] and Wang et al. [41] analyzed the source of particles in Beijing and Guangzhou, respectively, and obtained the contribution ratio of different human activities to the local PM_2.5_. Hou et al. [42] found that the main pollutants in the central and eastern regions of China played a decisive role in the spatial distribution and seasonal variation of PM_2.5_.

There are two drawbacks in the above studies. First, the effects of human activities on PM_2.5_ in different regions would have the same characteristics, which mainly focus on the economic and social development, transportation, energy utilization, etc. Second, while these studies can provide certain references for PM_2.5_ pollution reduction, such studies fail to incorporate the meteorological factors with human activities in the PM_2.5_ investigation.

Meteorological events and human activities are two categories of main contributing factors of PM_2.5_. At present, major measures of the PM_2.5_ pollution include the PM_2.5_ concentration in the atmosphere and days of PM_2.5_ pollution. Since the current haze weather has a great impact on people’s lives and physical health, and the haze weather is mainly caused by PM_2.5_ pollution, this study mainly considered the number of pollution days with PM_2.5_ as the primary pollutant, instead of the number of pollution days with SO_2_, NO_X_, CO, O_3_, PM_10_ and other pollution factors as the primary pollutant.

With the regard of the above considerations, the purpose of our study was to investigate the effect of meteorological factors and human activities on the local PM_2.5_ pollution in 2013–2016. Selected meteorological factors include wind speed, precipitation, temperature, atmospheric pressure, relative humidity. Human activities selected contain economic development, social progress, transportation and energy utilization. The research variables are the above influencing factors, and the areas in the study include 13 cities in Jiangsu Province.

The remainder of this paper is structured as follows: Section 2 selects indicator and describe data sources. Section 3 develops the chance-constrained stochastic DEA model. In Section 4, we apply the stochastic DEA to the selected 13 cities in Jiangsu Province at different risk levels for the years 2013 to 2016, and then analyze and classify the results. In Section 5, summary results are presented, along with a comparative analysis of analytical results of Section 4. Section 6 draws the conclusions and proposes some countermeasures.

## 2. Indicator Selection and Data Sources

This section selects meteorological factors and human activities as the study variables. Moreover, data sources will be illustrated in this section.

### 2.1. Indicator Selection

The input variables and output variable are selected in this section.

#### 2.1.1. Meteorological Factors

As for the selection of meteorological factors, many studies have adopted daily average or hourly monitoring values of meteorological factors [8,9,10,15,16,17,18,19,20,21,22,23,24,25,26,27], however, there is no uniform meteorological factors selection standards. Since meteorological factors may not affect the formation of PM_2.5_ pollution days until they are over or below a critical value, it is inappropriate to study the relationship between the monitoring mean of meteorological factors and PM_2.5_ pollution days.

For wind speed, only wind conditions with breeze or no wind are conducive to PM_2.5_ agglomeration. For precipitation, no precipitation day or no effective precipitation day is conducive to PM_2.5_ agglomeration. For temperature, the temperature decreases will generally be accompanied by a large wind speed, which is not conducive to PM_2.5_ agglomeration, and temperature rises will generally be accompanied by a calm weather, which is conducive to PM_2.5_ agglomeration. For air pressure, air pressure increase, due to the warming of high-pressure center, is conducive to the accumulation of PM_2.5_, whereas the reduction of air pressure is not conducive to PM_2.5_ agglomeration. For relative humidity, lower (less than 20%) or higher (greater than 90%) relative humidity, either too dry or too wet, is not conducive to the accumulation of contaminants.

In general, it is the interaction of various meteorological factors that affects the PM_2.5_ concentration. We can use the nodes where the meteorological factors affect PM_2.5_ as a “disaster point” (meteorological conditions in favor of the formation of PM_2.5_ such as wind speed < 1.5 m/s, no precipitation, temperature rising, air pressure drops, relative humidity between 60% and 90% etc.), with the “days” data of these nodes as disaster data, to study the relation between meteorological factors “disaster point” days and PM_2.5_ pollution days. At the same time, the data distribution characteristics of meteorological factors will be incorporated in the study.

Based on the above ideas, this study combines the data distribution characteristics of 5 meteorological factors in Jiangsu Province, wind speed < 1.5 m/s, no precipitation day, positive temperature change (current day temperature minus the previous day temperature is greater than 0), negative pressure change (current day pressure minus the previous day pressure is less than 0), and relative humidity (60–90%, excluding precipitation days). The days at the five meteorological nodes are used as research data. See Table 1. Using Grey Correlation Analysis, the study investigated the relationship between disaster point days and PM_2.5_ pollution days. The results are shown in Appendix A, Table A1.

#### 2.1.2. Human Activities

In 2015, Jiangsu Province launched the PM_2.5_ source analysis work. Based on the emission source list method, the source analysis results of Nanjing is displayed in [43]: coal burning contributed 27.4%, industrial production contributed 19.0%, vehicles exhaust contributed 24.6%, dust extraction contributed 14.1%, other pollution sources contributed 14.9%. According to the data, coal consumption is the biggest pollution source of air pollution in Nanjing.

Changzhou and Nantong had successively announced the results of PM_2.5_ source analysis. The source analysis result of Changzhou is published in [44]: the industrial production process accounted for the highest proportion, 25%, coal burning accounted for 23%, dust extraction accounted for 22%, automobile and diesel vehicle exhaust, non-road machinery and other mobile source emissions accounted for 22%, other sources of pollution accounted for 8%.

The source analysis result of Nantong display [45]: coal combustion accounted for 26%, mobile sources accounted for 24%, industrial production and dust generation account for 23% and 18%, respectively.

In addition, the statistical analysis of the highest and lowest concentrations of PM_2.5_ in 13 cities in Jiangsu Province in summer and winter 2016 shows that human activities have a much higher influence on PM_2.5_ concentration in winter than in summer due to the influence of meteorological factors such as more stable weather and poor diffusion conditions in winter.

According to the PM_2.5_ sources analysis results, PM_2.5_ sources are related to industrial development, energy utilization, transportation, social progress etc. Hence, we choose five groups of indicators, industrial development, social progress, transportation, energy utilization and ecological protection, as input variables for human activities.

Among them, the industrial development group selected “gross output value of industrial enterprises above designated size” as input variable. The social progress group selected “urbanization rate, population density, building construction area” as input variables. The transportation group selected “civil car ownership, number of public transportation vehicles under operation” as input variables. The energy utilization group selected “energy consumption of per 10,000 yuan industrial cross output value, total coal consumption” as input variables. The ecological protection group selected “green coverage rate of built-up areas” as input variable. The input variables and the output variable are summarized in Table 2.

### 2.2. Data Sources

Meteorological data was derived from the daily meteorological data of China Meteorological Data Network, which is an authoritative and unified sharing service platform for China meteorological administration to open meteorological data resources to domestic and global users. the network portal is http://data.cma.cn., from 2013 to 2016. According to the selection and treatment of “disaster point” set by this study, quantitative information on the data regarding days of “disaster point” for the five meteorological factors was obtained.

The PM_2.5_ data comes from the daily monitoring data of the atmospheric pollutants of Environmental Protection Department of Jiangsu Province, which is the functional department of Jiangsu Province, responsible for establishing and improving the basic system of environmental protection, environmental monitoring and information release, etc., from 2013 to 2016. In this study, based on the air quality index (AQI) and PM_2.5_ average daily concentration, the day, in which the air quality index was lightly polluted and above, and the 24-h average concentration of PM_2.5_ exceeds 75 μg/m^3^, was selected. The human activities data come from the statistical yearbooks of Jiangsu Province and 13 cities from 2014 to 2017.

## 3. Model Construction

Based on the multilayer perceptron neural network (NPNN), clustering algorithm, multiple linear regression (MLR), random forest regression (RFR), and so on, the identification and source analysis of PM_2.5_ influencing factors are the focus of current attention [46,47,48]. By incorporating the data distribution characteristics of meteorological factors and human activities, it is helpful to explore the essential features of data in a more real and comprehensive way. This study uses two-dimensional data of means and variances for modeling, which is difficult to handle using conventional methods.

Stochastic DEA is an extension of the DEA method, and its input and output variables are characterized by randomness describing the interference of measurement error, data noise and other random factors, reflecting the reality where observed data may deviate from true values due to random sampling [49]. Therefore, the stochastic DEA has a great advantage in dealing with performance evaluation in uncertain environment, especially in random input and output environment [50]. The first study on stochastic DEA was Sengupta [51], he used the reliability function to calculate the efficiency of the random input and output system. In order to make the solution of the stochastic DEA model deterministic, the constraint condition is added to the stochastic DEA model and transformed into a chance-constrained stochastic DEA model, which requires DEA to be valid at a certain confidence level 1 − α (0 < α < 1).Bruni et al. [52] proposed a stochastic DEA model based on joint probabilistic constraints. Cooper et al. [53] studied the stochastic DEA opportunity constraint model with random input-output data and discussed the deterministic equivalence form of the model. At the same time, they also discussed the sensitivity analysis in the case that only the data of the evaluated unit was random. Based on the study by Cooper et al. [53], Khodabakhshi [54,55] studied the super efficiency of stochastic DEA model in the form of opportunistic constrained programming from the perspectives of output and input respectively.

Chance constrained DEA breaks the rigid constraints of the traditional DEA model on inputs and outputs of decision units, allowing the evaluation unit to exceed the front edge under the given probability constraint, which is generally set statistically to some small enough confidence level [56]. Compared with other methods, the chance constrained stochastic DEA has certain advantages. For example, it has no requirements on sample size and index correlation, and it is more reasonable to use this method for data with large sample size, uncertainty and only general distribution characteristics.

Therefore, the chance constrained stochastic DEA model that considers the data distribution characteristics is introduced into this study to explore the influencing factors of PM_2.5_ pollution in different regions where uncertainty condition exists.

The DEA method is usually a measure of efficiency, while this study introduced the chance constrained stochastic DEA into the study of PM_2.5_ influencing factors, which is a promotion of the application field of this method.

Suppose there are *n* Decision Making Units (DMUs). In this study, *n* = 13, representing the 13 prefecture-level cities in Jiangsu Province. There are *m* different input variables:

(1) $${\tilde{x}}_{ij}\left(i=1,2,\cdots ,m\right)$$

In this study, *m* = 14, representing the 14 input variables. There are *s* different outputs *ỹ_rj_* (*r* = 1, 2, ··*s*). In this study, *s* = 1, denoting the output variable. Each DMU_j_ (*j* = 1, 2, ··*n*) includes *m* different input variables and s different output variables.

Input and output variables of each DMU are random vectors, the corresponding means are:

(2) $${\bar{x}}_{ij}\left(i=1,2,\cdots ,m\right)$$

and:

(3) $${\bar{y}}_{rj}\left(r=1,2,\cdots ,s\right)$$

Assume that the DMU being evaluated is DMU_o_:

(4) o∈{1, 2,…,n}

The chance-constrained stochastic DEA model based on different risk levels is the following:

O*bjective function*: Max *θ_o_*

(5) $$\mathrm{Max}\;\theta_{o}$$

*Constraints*:

(6) $$\left\{ \begin{array}{l} \sum_{j=1}^{n}\lambda_{j}{\bar{x}}_{ij}-\Phi^{-1}\left(\alpha \right)\sqrt{\sum_{j\neq o}\lambda_{j}^{2}{\left(\sigma_{ij}^{I}\right)}^{2}+{\left(\lambda_{o}-1\right)}^{2}{\left(\sigma_{io}^{I}\right)}^{2}}\leq {\bar{x}}_{io},i=1,\;2,\ldots ,m\\ \sum_{j=1}^{n}\lambda_{j}{\bar{y}}_{rj}+\Phi^{-1}\left(\alpha \right)\sqrt{\sum_{j\neq o}\lambda_{j}^{2}{\left(\sigma_{rj}^{o}\right)}^{2}+{\left(\lambda_{o}-\theta_{o}\right)}^{2}{\left(\sigma_{ro}^{o}\right)}^{2}}\geq \theta_{o}{\bar{y}}_{ro},r=1,\;2,\ldots ,s\\ \sum_{j=1}^{n}\lambda_{j}=1\\ \lambda_{j}\geq 0,j=1,\;2,\ldots ,n \end{array}\right.$$

According to Lan [50], in Equation (5), *θ_o_* is the target object function to be optimized. In Equation (6), α ∈ [0, 1] is the risk level (or significance level), that is, the risks faced in decision-making. A correct decision leads to a lower risk, and a wrong decision results in a higher risk. In our study, the higher the risk level, the higher the probability of PM_2.5_ pollution days after the management decision is made.

Φ^−1^ (α) is the value of the inverse distribution function of the standard normal distribution function at α; *σ^I^_ij_* and *σ^0^_ij_* are the standard deviations of:

$${\tilde{x}}_{ij}\;\mathrm{and}\;{{\tilde{y}}_{rj}}_{j},\;\mathrm{respectively}$$

*λ_j_* is the parameter of *DMU_j_*;

$$\Phi^{-1}\left(\alpha \right)\sqrt{\sum_{j\neq o}\lambda_{j}^{2}{\left(\sigma_{ij}^{I}\right)}^{2}+{\left(\lambda_{o}-1\right)}^{2}{\left(\sigma_{io}^{I}\right)}^{2}}$$

involves the standard deviations of input variables and different values of α are used here to study the change of the target optimal solution *θ_o_* at different α levels:

$$\Phi^{-1}\left(\alpha \right)\sqrt{\sum_{j\neq o}\lambda_{j}^{2}{\left(\sigma_{rj}^{o}\right)}^{2}+{\left(\lambda_{o}-\theta_{o}\right)}^{2}{\left(\sigma_{ro}^{o}\right)}^{2}}$$

involves the standard deviations of output variables and different values of α are used here to study the change of the target optimal solution *θ_o_* at different α levels. The reciprocal of *θ_0_* is the stochastic efficiency of DMU_0_.

## 4. Results and Analyses

### 4.1. Stochastic DEA Results for 2013–2016

**Figure 1:**
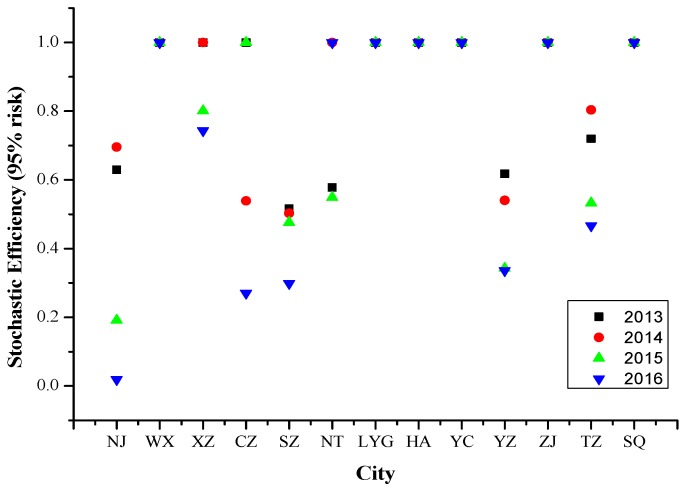
Stochastic efficiency values of 13 cities in Jiangsu Province at 95% risk level from 2013 to 2016. Abbreviations: Nanjing (NJ), Wuxi (WX), Xuzhou (XZ), Changzhou (CZ), Suzhou (SZ), Nantong (NT), Lianyungang (LYG), Huai’an (HA), Yancheng (YC), Yangzhou (YZ), Zhenjiang (ZJ), Taizhou (TZ), Suqian (SQ).

Referring to Figure 1, at a 95% risk level, Wuxi, Lianyungang, Huai’an, Yancheng, Zhenjiang and Suqian were the cities with stochastic DEA efficiency from 2013 to 2016. It indicates that the prevention and control of PM_2.5_ pollutions in these cities was relatively effective in recent years.

Before the result analysis, it should be noted that the analysis process is like the evaluation results obtained from 2013 to 2016. In view of space constraint, this study took 2013 as an example to give detailed analysis process and conclusions on stochastic DEA results of 13 cities. For the assessment results from 2014 to 2016, this study only provided a comprehensive conclusion. In addition, the random efficiency values obtained from 2014 to 2016 were shown in Appendix A, Table A2, Table A3, Table A4, Table A5, Table A6 and Table A7.

#### 4.1.1. Year 2013

At the 95% risk level, the efficiency values of Wuxi, Xuzhou, Changzhou, Lianyungang, Huai’an, Yancheng, Zhenjiang and Suqian were 1, and the remaining cities were ranked by efficiency values (from high to low) as follows Taizhou, Nanjing, Yangzhou, Nantong and Suzhou. But at 50% risk level or less, the efficiency values of all 13 cities were 1.

**Figure 2:**
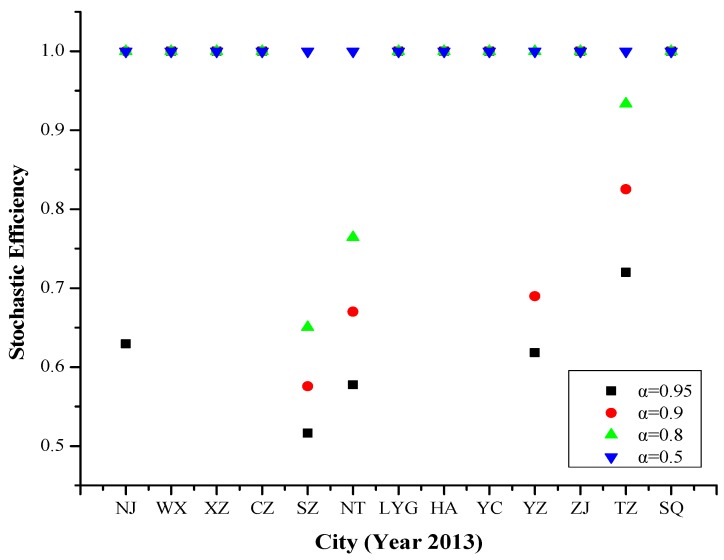
Stochastic efficiency values of 13 cities in Jiangsu Province at different risk levels in 2013.

Referring to Figure 2, the efficiency values of Nanjing, Suzhou, Nantong, Yangzhou and Taizhou changed with the risk level increase, and their efficiency values decreased as the risk level increased. It shows that in 2013, PM_2.5_ pollution days in these cities were greatly affected by meteorological factors and human activities.

In order to investigate the relationship between input variables and the output variable, firstly this study obtained the relevant efficiency values by deleting grouping variables. As the risk levels are between 0.05 and 0.5, after deleting the grouping variables, the efficiency values of DMUs had not changed; When the risk level is 0.8, 0.9 and 0.95, the DMUs whose stochastic efficiency values changed by deleting grouping variables are shown and analyzed in Table 3.

To investigate the relationship between different input variables and the output variable, this study further derives the efficiency values of DMUs by deleting single input variable. The number of cities which stochastic efficiency values changed by deleting single input variable in diffident risk levels in 2013 is shown in Table 4, and the analyses results are shown in Table 5.

In general, as the risk level increased, cities with efficiency values of 1 decreased in 2013. At the 95% risk level, only eight cities had efficiency values of 1. The PM_2.5_ pollution days in most cities were dominated by meteorological factors and social progress, and few cities were affected by transportation, energy utilization, ecological protection. The main factors affecting PM_2.5_ pollution days were wind speed, relative humidity, urbanization rate, population density, building construction area, number of public transportation vehicles under operation, energy consumption of per 10,000 yuan industrial cross output value, total coal consumption and green coverage rate of built-up areas.

#### 4.1.2. Year 2014

**Figure 3:**
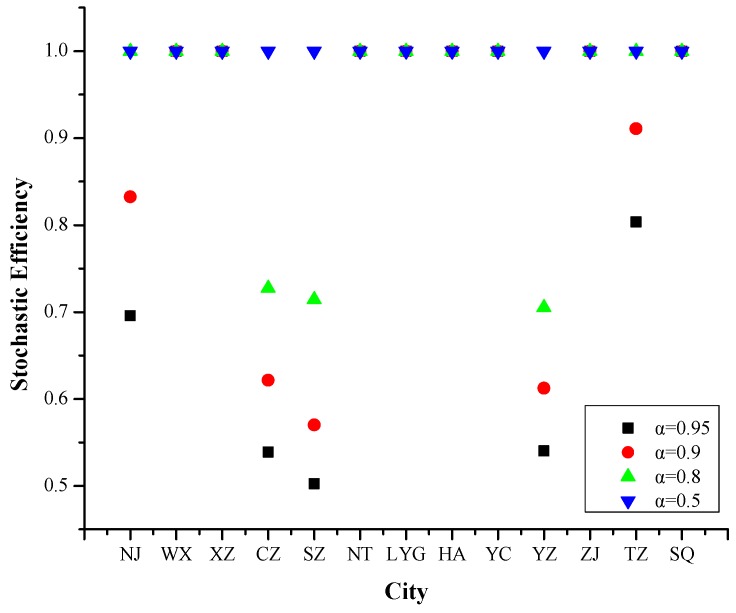
Stochastic efficiency values of 13 cities in Jiangsu Province at different risk levels in 2014.

Referring to Figure 3, the efficiency values of Nanjing, Changzhou, Suzhou, Yangzhou and Taizhou changed with the increased risk levels.

In general, the results in 2014 were similar to in 2013. But the specific influencing factors on PM_2.5_ pollution days were a little different compared with 2013, which also included no precipitation day and negative pressure change but did not included urbanization rate.

#### 4.1.3. Year 2015

**Figure 4:**
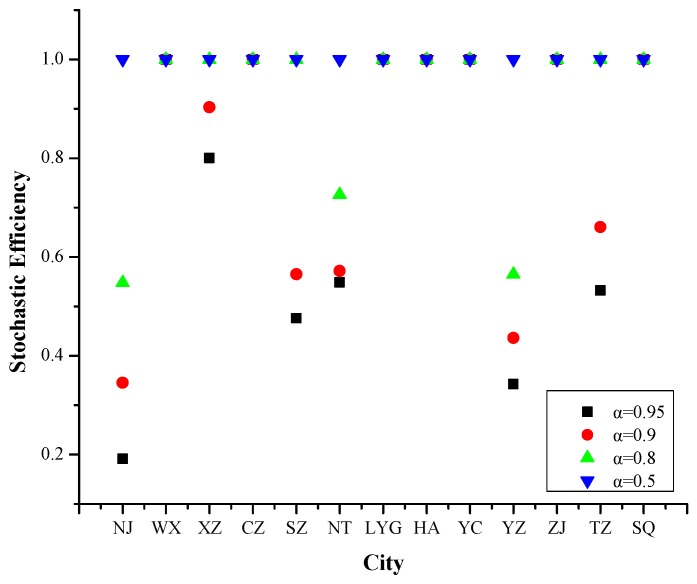
Stochastic efficiency values of 13 cities in Jiangsu Province at different risk levels in 2015.

Refer to Figure 4, the efficiency values of Nanjing, Xuzhou, Suzhou, Nantong, Yangzhou and Taizhou changed with the increased risk levels.

Compared to 2013-2014, cities with efficiency values of 1 in 2015 have decreased. At the 95% risk level, only 7 cities have stochastic efficiency values of 1. The PM_2.5_ pollution days in most cities were dominated by meteorological factors and social progress, but unlike 2013–2014, the PM_2.5_ pollution days in Nanjing were also affected by industrial development. The specific influencing factors in 2015 were wind speed, no precipitation day, relative humidity, population density, building construction area, number of public transportation vehicles under operation, total coal consumption and green coverage rate of built-up areas.

#### 4.1.4. Year 2016

**Figure 5:**
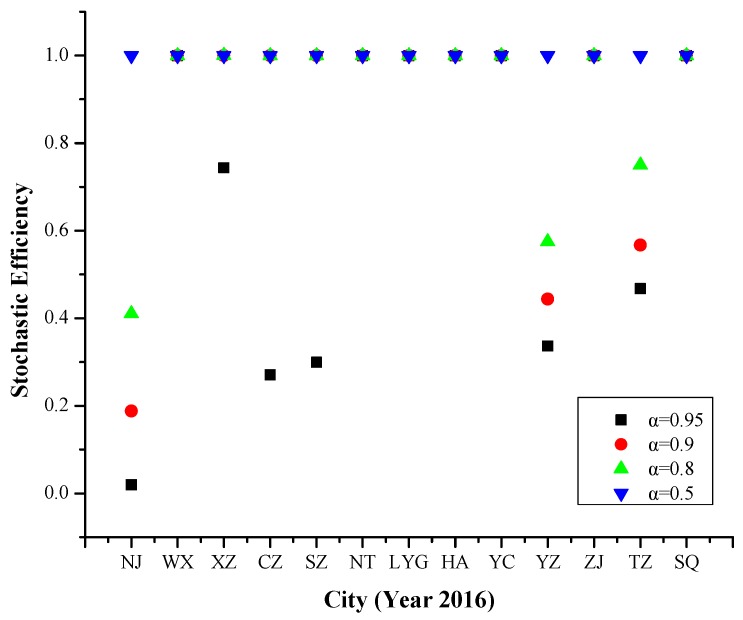
Stochastic efficiency values of 13 cities in Jiangsu Province at different risk levels in 2016.

Referring to Figure 5. The efficiency values of Nanjing, Xuzhou, Changzhou, Suzhou, Yangzhou and Taizhou changed with the increased risk levels.

In 2016, the results were similar to in 2015. But the specific influencing factors on PM_2.5_ pollution days were a little different compared with 2015, which also included positive temperature change, and the cities affected by various factors had also increased.

### 4.2. Regional Stochastic DEA Results

According to the economic development, geographical location and other factors, the selected 13 cities in Jiangsu Province are divided into three regions: Southern Jiangsu Province, Central Jiangsu Province and Northern Jiangsu Province. The geographical areas can also be divided into coastal and inland area. Among them, Southern Jiangsu Province includes Nanjing, Zhenjiang, Suzhou, Wuxiand Changzhou. Central Jiangsu Province includes Yangzhou, Taizhou and Nantong. Northern Jiangsu Province includes Xuzhou, Lianyungang, Huai’an, Yancheng and Suqian. Coastal area includes Nantong, Lianyungang and Yancheng. Inland area includes ten cities, Nanjing, Xuzhou, Changzhou, Suzhou, Yangzhou, Taizhou, Wuxi, Zhenjiang, Huai’an and Suqian.

To examine the relationship between input variables and output variable among cities of different regions, the stochastic efficiency values obtained by deleting grouping variables and deleting single input variables were sorted by regions (see Appendix A, Table A8, Table A9, Table A10, Table A11, Table A12, Table A13, Table A14, Table A15, Table A16 and Table A17) to further analyze and evaluate the common and individual characteristics of PM_2.5_ pollution for different regions. In view of the space limitation, the detailed analysis process is no longer listed, but the comprehensive conclusions of regional analysis are provided.

#### 4.2.1. Southern Jiangsu Province

**Figure 6:**
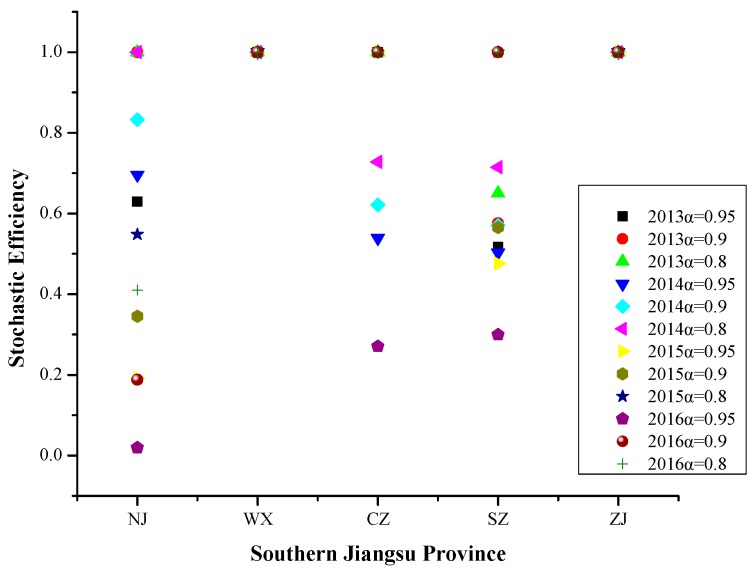
Stochastic efficiency values in southern Jiangsu Province at different risk levels in 2013–2016.

From 2013 to 2016, referring to Figure 6, the efficiency values of Wuxi and Zhenjiang were 1 at different risk levels. The efficiency values of Nanjing, Changzhou and Suzhou showed a monotonous non-increasing trend as the risk level increase.

The results obtained by deleting grouping input variables and deleting single input variables are summarized as follows:

From 2013 to 2016, the efficiency values of Wuxi and Zhenjiang were 1 at different risk levels, which indicates that the two cities have relatively high levels of particulate pollution control. The efficiency values of Nanjing, Changzhou and Suzhou showed a monotonous non-increasing trend as the risk level increases. The PM_2.5_ pollution days in Southern Jiangsu Province were dominated by meteorological factors and social progress, less affected by industrial development, transportation, and energy utilization. Due to the high level of urbanization, dense population and advanced industrial pollution control, energy utilization and traffic management in Southern Jiangsu Province, besides meteorological factors, social progress is an important factor affecting PM_2.5_ pollution in recent years. The specific influencing factors were wind speed, relative humidity, population density, building construction area, total coal consumption and green coverage rate of built-up areas.

#### 4.2.2. Central Jiangsu Province

**Figure 7:**
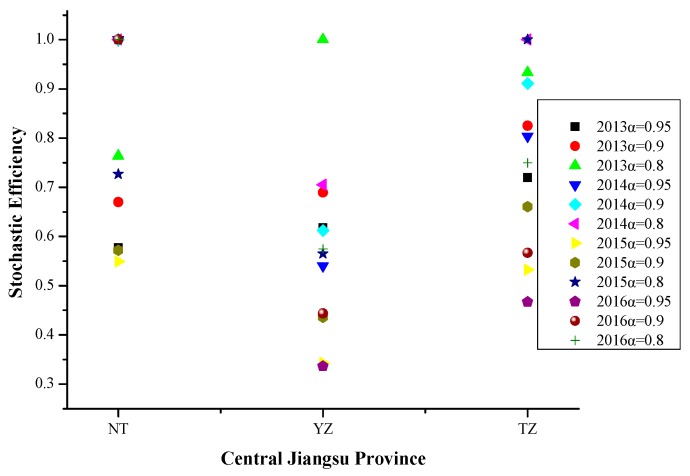
Stochastic efficiency values in central Jiangsu Province at different risk levels in 2013–2016.

Referring to Figure 7, At different risk levels, the Nantong’s efficiency values were 1 in 2014 and 2016. In Yangzhou and Taizhou, the stochastic efficiency showed a monotonous non-increasing trend as the risk level increases.

From 2013 to 2016, PM_2.5_ pollution days in Central Jiangsu Province were affected by many factors, such as meteorological factors, social progress, transportation, energy utilization, but have little to do with the industrial development. Since Central Jiangsu Province is inferior to Southern Jiangsu Province in energy utilization and traffic management, the two factors become important factors of PM_2.5_ pollution in Central Jiangsu Province. The specific influencing factors were wind speed, no precipitation day, positive temperature change, negative pressure change, relative humidity, population density, civil car ownership, number of public transportation vehicles under operation, energy consumption of per 10,000 yuan industrial cross output value, total coal consumption and green coverage rate of built-up areas.

#### 4.2.3. Northern Jiangsu Province

**Figure 8:**
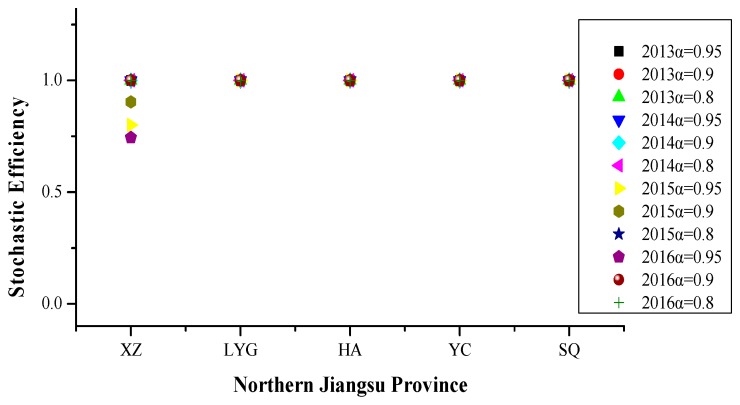
Stochastic efficiency values in northern Jiangsu province at different risk levels in 2013–2016.

Referring to Figure 8, From 2013 to 2016, the efficiency values of Lianyungang, Huai’an, Yancheng and Suqian were 1 at different risk levels. With the increase of risk level, Xuzhou’s efficiency value presented a monotonous and non-increasing trend. In 2013–2016, PM_2.5_ pollution days in Northern Jiangsu Province were more affected by social progress, followed by meteorological factors and transportation, and the impact of industrial development, energy utilization and ecological protection was minimal.

This indicates that Northern Jiangsu Province is in a period of rapid urbanization and population growth, and the number of motor vehicles increases sharply. Therefore, in addition to meteorological factors, social progress and motor vehicles become important factors affecting PM_2.5_ pollution in Northern Jiangsu Province. The specific influencing factors were relative humidity, population density and civil car ownership.

#### 4.2.4. Coastal Area

**Figure 9:**
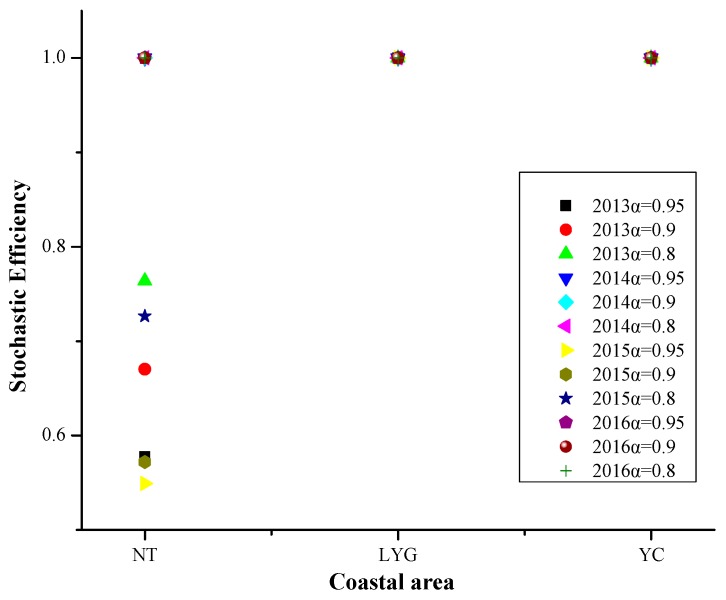
Stochastic efficiency values in coastal area at different risk levels in 2013–2016.

Referring to Figure 9, From 2013 to 2016, the efficiency values of Lianyungang and Yancheng were 1 at different risk levels. Nantong only had efficiency values of 1 in 2014 and 2016.

From 2013 to 2016, the PM_2.5_ pollution days in Nantong was largely affected by the meteorological factors and social progress at different risk levels, Lianyungang and Yancheng were mainly affected by social progress. The main factors affecting the PM_2.5_ pollution days in Nantong were wind speed, no precipitation day, positive temperature change, population density, number of public transportation vehicles under operation, and energy consumption of per 10,000 yuan industrial cross output value. The main factors affecting the PM_2.5_ pollution days in Lianyungang and Yancheng were population density and building construction area.

#### 4.2.5. Inland Area

**Figure 10:**
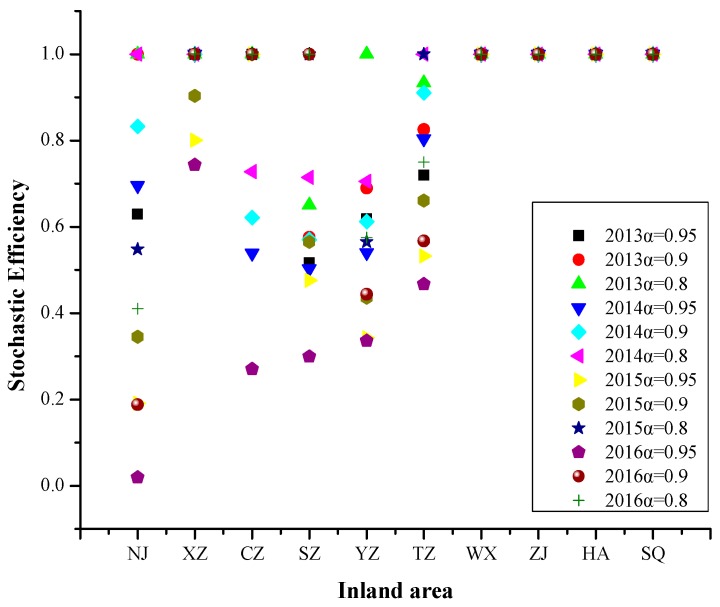
Stochastic Efficiency Values in Inland Area at Different Risk Levels in 2013–2016.

Referring to Figure 10, In 2013–2016, the efficiency values of Wuxi, Zhenjiang, Huai’an and Suqian were 1 at different risk levels. The efficiency Values of other cities changed more complex at different risk levels.

From 2013 to 2016, the PM_2.5_ pollution days in inland area was affected by meteorological factors and social progress at different risk levels, followed by transportation, energy utilization, and ecological protection, less affected by industrial development. The PM_2.5_ pollution days in inland area were related to most of input variables, in addition to the two input variables of “gross output value of industrial enterprises above designated size” and “urbanization rate”.

## 5. Results Comparison

We summarized the analysis results of the fourth part and drew conclusions of generality and personality, with the results shown in Table 6.

## 6. Conclusions and Policy Recommendations

### 6.1. Conclusions

PM_2.5_ is mainly produced by human activities, but its migration, as well as the formation in some cases, is largely driven by meteorological factors. This study aimed at the influencing factors of PM_2.5_ in different regions. We adopted the chance constrained stochastic DEA model, took meteorological factors and human activities as input variables, and PM_2.5_ pollution days as output variables. By deleting grouping input variables and single input variable, we study the stochastic efficiency values of 13 cities in Jiangsu Province under different risk levels. If one or a grouping input variables was deleted and the stochastic efficiency value of the DMUs changed, it is considered that the deleteing input variable or the grouping input variables had an impact on PM_2.5_ pollution day, and the influencing factors in different regions were sorted out from 2013 to 2016.

It is concluded that there were generality and personality factors of PM_2.5_ pollution for the selected 13 cities in Jiangsu Province. From the perspective of time series, cities affected by NPD, PTC, GOVIE, PD and CCO variables in 13 cities in Jiangsu Province increased, while cities affected by WS, NPC and UR variables decreased from 2013 to 2016. From the perspective of the subregion, the number of PM_2.5_ pollution days in southern Jiangsu Province was greatly affected by meteorological factors and social progress, but less affected by industrial development, transportation and energy utilization. The number of PM_2.5_ pollution days in central Jiangsu Province was affected by meteorological, social progress, transportation, energy utilization, but it had little relationship with industrial development. In the northern Jiangsu Province, the number of PM_2.5_ pollution days was greatly affected by social progress, followed by meteorological factors and transportation, and the least affected by industrial development, energy utilization and ecological protection. In coastal area, the number of PM_2.5_ pollution days in Nantong city was greatly affected by meteorological factors and social progress, while Lianyungang and Yancheng were only greatly affected by social progress. In inland area, the number of PM_2.5_ pollution days was largely affected by meteorological factors and social progress, followed by transportation, energy utilization and ecological protection, and less affected by industrial development.

In addition, the evaluation model adopted in this study has the following characteristics:

(1) Consider the distribution characteristics of data.
(2) Comprehensively investigate the meteorological factors and human activities.
(3) Differentiate multiple effective decision units.

The chance constrained stochastic DEA focuses on processing large sample data, especially panel data with incomplete data, incomplete information and only general distribution. This method pays attention to the input-output relationship between variables, that is, efficiency, so the stochastic DEA and other similar techniques can also be used for environmental performance, environmental efficiency, energy efficiency and other evaluation studies in terms of environmental sciences.

### 6.2. Policy Recommendations

First, the cities in Jiangsu Province should pay attention to the impacts of meteorological conditions on local PM_2.5_ pollution and intensify haze forecasting and early warning. At the same time, each city should comprehensively consider the diffusion or agglomeration effects of pollutants under different meteorological conditions. When formulating management policies, timely selects measures to prevent and reduce haze pollution caused by adverse meteorological conditions. Second, cities should reach consensus and strengthen regional joint defense and control. Haze pollution often has regional and compound characteristics. The neighboring cities should strengthen the regional joint prevention and control, jointly formulate and implement the joint control measures for air pollution and co-improve the regional air quality.

The limitation is that this is a qualitative study on the factors affecting PM_2.5_. The prevention and control of PM_2.5_ pollution need to maintain a continuous long-term effort. Future research can further explore the reason of invalid stochastic efficiency and investigate deeper relationship between PM_2.5_ pollutions, meteorological factors and human activities. For example, study the impact of cooperation and competition in different regions on PM_2.5_ pollution, in order for providing useful reference and support for local environmental protection measures.

## Figures and Tables

**Table 1:** The highest and lowest concentrations of PM_2.5_ in summer and winter in 13 cities in Jiangsu Province in 2016.

**Concentration of PM_2.5_**	**Nanjing**		**Wuxi**		**Xuzhou**		**Changzhou**		**Suzhou**		**Nantong**		**Lianyungang**	
	**S**	**W**	**S**	**W**	**S**	**W**	**S**	**W**	**S**	**W**	**S**	**W**	**S**	**W**
Maximum concentration of PM_2.5_	98	184	85	161	77	282	66	171	69	163	66	159	52	211
Minimum concentration of PM_2.5_	10	15	11	23	13	16	12	24	10	23	10	18	9	13
**Concentration of PM_2.5_**	**Huai’an**		**Yancheng**		**Yangzhou**		**Zhenjiang**		**Taizhou**		**Suqian**			
	**S**	**W**	**S**	**W**	**S**	**W**	**S**	**W**	**S**	**W**	**S**	**W**		
Maximum concentration of PM_2.5_	73	206	67	216	76	187	68	187	90	185	67	218		
Minimum concentration of PM_2.5_	13	19	6	16	13	23	8	20	11	18	12	23		

Note: Abbreviations-Summer (S), Winter (W).

**Table 2:** Research variables.

Research Variables	Grouping Variable (Abbreviation)	Single Input Variable	Abbreviation	Unit
Input variables	Meteorological Factors (MF)	Wind Speed (< 1.5 m/s)	WS	days
		No Precipitation Day	NPD	
		Positive Temperature Change	PTC	
		Negative Pressure Change	NPC	
		Relative Humidity (60–90%, excluding precipitation days)	RH	
	Industrial Development (ID)	Gross Output Value of Industrial Enterprises above Designated Size	GOVIE	hundred million
	Social Progress (SP)	Urbanization Rate	UR	%
		Population Density	PD	people per square kilometer
		Building Construction Area	BCA	Ten thousand square meters
	Transportation (T)	Civil Car Ownership	CCO	Ten thousand cars
		Number of Public Transportation Vehicles under Operation	NPTVO	Standard number
	Energy Utilization (EU)	Energy Consumption of per 10,000 Yuan Industrial Cross Output Value	EC	Ton of standard coal per ten thousand yuan
		Total Coal Consumption	TCC	Ton of standard coal
	Ecological Protection (EP)	Green Coverage Rate of Built-up Areas	GCRBA	%
Output variable	Haze Pollution	PM_2.5_ Pollution Days	-	days

Note: Rail transit is not included in number of public transportation vehicles under operation.

**Table 3:** The number of cities which stochastic efficiency values changed by deleting single input variable in diffident risk levels from 2013 to 2016.

**DMUs**	**Delete WS**	**Delete NPD**	**Delete PTC**	**Delete NPC**	**Delete RH**	**Delete GOVIE**	**Delete UR**
2013	5	1	1	0	6	0	1
2014	4	3	1	2	3	0	0
2015	4	4	0	0	6	1	0
2016	4	5	2	0	5	1	0
**DMUs**	**Delete PD**	**Delete BCA**	**Delete CCO**	**Delete NPTVO**	**Delete EC**	**Delete TCC**	**Delete GCRBD**
2013	3	2	1	2	2	2	2
2014	3	4	1	3	1	2	2
2015	5	5	1	2	1	1	2
2016	4	2	2	3	2	2	2

**Table 4:** The number of cities which stochastic efficiency values changed by deleting single input variable in diffident risk levels from 2013 to 2016.

**DMUs**	**Delete WS**	**Delete NPD**	**Delete PTC**	**Delete NPC**	**Delete RH**	**Delete GOVIE**	**Delete UR**
2013	5	1	1	0	6	0	1
2014	4	3	1	2	3	0	0
2015	4	4	0	0	6	1	0
2016	4	5	2	0	5	1	0
**DMUs**	**Delete PD**	**Delete BCA**	**Delete CCO**	**Delete NPTVO**	**Delete EC**	**Delete TCC**	**Delete GCRBD**
2013	3	2	1	2	2	2	2
2014	3	4	1	3	1	2	2
2015	5	5	1	2	1	1	2
2016	4	2	2	3	2	2	2

**Table 5:** The result analysis of deleting single input variables in 2013.

Deleting Single Variable	Risk Level	City with Changing Value	Result Analysis
Delete WS	α = 0.95	Nanjing, Changzhou, Nantong and Taizhou	The wind speed (<1.5 m/s) of these cities was related to the local haze pollution occurrence. Stable weather with low wind speed is not conducive to the diffusion of pollutants, thus exacerbating the formation of pollution days.
	α = 0.9	Nanjing, Wuxi, Nantong and Taizhou	
	α = 0.8		
Delete NPD	α = 0.95	-	The no precipitation day affected the PM_2.5_ pollution days in Nantong.
	α = 0.9	Nantong	
	α = 0.8	-	
Delete PTC	α = 0.95	-	The positive temperature change affected the PM_2.5_ pollution days in Nantong.
	α = 0.9	-	
	α = 0.8	Nantong	
Delete RH	α = 0.95	Nanjing, Wuxi, Xuzhou, Changzhou, Suzhou and Yangzhou	When the relative humidity of these cities is between 60 and 90%, and no precipitation, there is a greater chance of haze pollution.
	α = 0.9	Wuxi, Xuzhou, Suzhou and Yangzhou	
	α = 0.8	Wuxi, Xuzhou, Changzhou and Yangzhou	
Delete UR	α = 0.95	Nantong	The urbanization rate in Nantong has impact on the local PM_2.5_ pollution days.
	α = 0.9		
	α = 0.8		
Delete PD	α = 0.95	Suzhou, Huai’an and Yangzhou	The population density has impact on the PM_2.5_ pollution days in these cities.
	α = 0.9		
	α = 0.8		
Delete BCA	α = 0.95	Wuxi and Zhenjiang	The pollutions caused by the building construction area in the two cities had certain relationship with the local PM_2.5_ pollution days.
	α = 0.9	-	
	α = 0.8	-	
Delete CCO	α = 0.95	Huai’an	The civil car ownership has impact on the local PM_2.5_ pollution days in Huai’an.
	α = 0.9		
	α = 0.8		
Delete NPTVO	α = 0.95	Nantong and Taizhou	The bus operations in these cities were related to the local PM_2.5_ pollution days.
	α = 0.9		
	α = 0.8	-	
Delete EC	α = 0.95	Taizhou	The energy utilization of the two cities affected the local PM_2.5_ pollution days.
	α = 0.9		
	α = 0.8	Nantong and Taizhou	
Delete TCC	α = 0.95	Changzhou	The local coal consumption in these cities affected the local PM_2.5_ pollution days.
	α = 0.9	Yangzhou	
	α = 0.8		
Delete GCRBA	α = 0.95	-	-
	α = 0.9	Suzhou and Taizhou	The local greening situation in the two cities affected the local PM_2.5_ pollution days.
	α = 0.8		

Note: the NPC and GOVIE were deleted, no city’s value changed.

**Table 6:** Comparison of PM_2.5_ influencing factors.

Classification	Generality	Personality
Years	With the risk level decrease, the influencing factors of PM_2.5_ pollution days reduced.	With the risk level change, the specific factors affecting PM_2.5_ pollution days were different.
	2013–2016, the number of cities with values of 1 decreased, and the higher the risk level, the fewer cities the values were effective.	At 95% risk level, there were more cities’ PM_2.5_ pollution days affected by transportation in 2013–2014 than in 2015–2016.
	In 2013–2016, PM_2.5_ pollution days of 13 cities in Jiangsu Province were affected by meteorological factors and social progress.	Wind speed and relative humidity had a significant impact on PM_2.5_ pollution days in 2013–2014; no precipitation days had greater impact on PM_2.5_ pollution days in 2015–2016.
Areas	Stochastic DEA effective regional sorting: Northern Jiangsu Province, Southern Jiangsu Province, Central Jiangsu Province.	The stochastic efficiencies of Yangzhou and Taizhou in Central Jiangsu Province were invalid.
	The PM_2.5_ pollution days in Southern and Central Jiangsu Province were closely related to meteorological factors and social progress.	The PM_2.5_ pollution days in Northern Jiangsu Province were closely related to social progress.
	The PM_2.5_ pollution days in Southern and Central Jiangsu Province were affected by most of the input variables.	The PM_2.5_ pollution days in Northern Jiangsu Province is only related to relative humidity, population density and civil car ownership.
	The PM_2.5_ pollution days in coastal and inland area were affected by meteorological factors, social progress, transportation and energy utilization, less affected by industrial development.	The PM_2.5_ pollution days in inland area was also related to ecological protection.
	The specific factors affecting the PM_2.5_ pollution days in coastal and inland areas were wind speed, no precipitation day, relative humidity, and population density.	The factors affecting the PM_2.5_ pollution days in inland area also included: building construction area, civil car ownership, total coal consumption and green coverage rate of built-up areas.

## Appendix A

**Table A1:** Grey correlation between disaster point days and PM_2.5_ pollution days.

Disaster Point Days	2013	2014	2015	2016
wind speed (<1.5 m/s)	0.5967	0.5237	0.5955	0.6250
no precipitation day (= 0 mm)	0.8450	0.7868	0.7905	0.7684
positive temperature change (℃)	0.8108	0.8282	0.6977	0.6364
negative pressure change (hpa)	0.8545	0.8167	0.7033	0.6374
relative humidity (60–90%, excluding precipitation days)	0.6327	0.6760	0.6122	0.5788

**Table A2:** The changes of stochastic efficiency value by deleting grouping input variables in 2014 (α = 0.95).

DMUs	*a* = 0.95	Delete MF	Delete ID	Delete SP	Delete T	Delete EU	Delete EP
Nanjing	0.6957	0.6098					
Wuxi	1.0000			0.5222			
Xuzhou	1.0000	0.6689					
Changzhou	0.5389			0.5234	0.5361		
Suzhou	0.5025	0.5047		0.4561			0.4770
Nantong	1.0000	0.3982			0.4915		
Lianyungang	1.0000						
Huai’an	1.0000			0.5820	0.7774		
Yancheng	1.0000			0.3721			
Yangzhou	0.5401			0.5064			
Zhenjiang	1.0000			0.5987			
Taizhou	0.8035	0.7725			0.7753		0.7675
Suqian	1.0000						

**Table A3:** The changes of stochastic efficiency value by deleting grouping input variables in 2015 (α = 0.95).

DMUs	*a* = 0.95	Delete MF	Delete ID	Delete SP	Delete T	Delete EU	Delete EP
Nanjing	0.1912	0.1761					
Wuxi	1.0000			0.4562			
Xuzhou	0.8004						
Changzhou	1.0000	0.3855					
Suzhou	0.4761	0.4666		0.4909			0.4849
Nantong	0.5489	0.5007					
Lianyungang	1.0000			0.4622			
Huai’an	1.0000			0.4864			
Yancheng	1.0000			0.3725			
Yangzhou	0.3427	0.3393				0.3151	
Zhenjiang	1.0000			0.3984			
Taizhou	0.5322						0.5251
Suqian	1.0000						

**Table A4:** The changes of stochastic efficiency value by deleting grouping input variables in 2016 (α = 0.95).

DMUs	*a* = 0.95	Delete MF	Delete ID	Delete SP	Delete T	Delete EU	Delete EP
Nanjing	0.0195	0.0187					
Wuxi	1.0000			0.3156			
Xuzhou	0.7436	0.7186					
Changzhou	0.2706	0.2615				0.2499	
Suzhou	0.2991	0.2833		0.2641			0.2886
Nantong	1.0000	0.2984		0.3226		0.3226	
Lianyungang	1.0000						
Huai’an	1.0000			0.4609			
Yancheng	1.0000			0.3222			
Yangzhou	0.3362	0.3361		0.3424	0.3362	0.3217	
Zhenjiang	1.0000			0.1833			
Taizhou	0.4671	0.4581					
Suqian	1.0000						

**Table A5:** The changes of stochastic efficiency value by deleting single input variable in 2014 (α = 0.95).

**DMUs**	***a* = 0.95**	**Delete WS**	**Delete NPD**	**Delete PTC**	**Delete NPC**	**Delete RH**	**Delete GOVIE**	**Delete UR**
Nanjing	0.6957	0.6494				0.6098		
Wuxi	1							
Xuzhou	1					0.679		
Changzhou	0.5389							
Suzhou	0.5025	0.5047						
Nantong	1	0.3982						
Lianyungang	1							
Huai’an	1							
Yancheng	1							
Yangzhou	0.5401							
Zhenjiang	1							
Taizhou	0.8035	0.7725						
Suqian	1							
**DMUs**	***a* = 0.95**	**Delete PD**	**Delete BCA**	**Delete CCO**	**Delete NPTVO**	**Delete EC**	**Delete TCC**	**Delete GCRBD**
Nanjing	0.6957							
Wuxi	1		0.5222					
Xuzhou	1							
Changzhou	0.5389		0.5234		0.5361			
Suzhou	0.5025		0.4561					0.477
Nantong	1				0.4915			
Lianyungang	1							
Huai’an	1	0.6079		0.7774				
Yancheng	1	0.4315						
Yangzhou	0.5401	0.5064						
Zhenjiang	1		0.5987					
Taizhou	0.8035				0.7837			0.7675
Suqian	1							

**Table A6:** The changes of stochastic efficiency value by deleting single input variable in 2015 (α = 0.95).

DMUs	*a* = 0.95	Delete WS	Delete NPD	Delete PTC	Delete NPC	Delete RH	Delete GOVIE	Delete UR	Delete PD	Delete BCA	Delete CCO	Delete NPTVO	Delete EC	Delete TCC	Delete GCRBD
Nanjing	0.1912	0.1761													
Wuxi	1.0000									0.4562					
Xuzhou	0.8004														
Changzhou	1.0000					0.3920									
Suzhou	0.4761	0.4618	0.4842			0.4761			0.4757	0.4909					0.4849
Nantong	0.5489	0.5221	0.5364												
Lianyungang	1.0000									0.4622					
Huai’an	1.0000								0.4883						
Yancheng	1.0000								0.4026						
Yangzhou	0.3427		0.3393											0.3151	
Zhenjiang	1.0000									0.3984					
Taizhou	0.5322														0.5251
Suqian	1.0000														

**Table A7:** The changes of stochastic efficiency value by deleting single input variable in 2016 (α = 0.95).

DMUs	*a* = 0.95	Delete WS	Delete NPD	Delete PTC	Delete NPC	Delete RH	Delete GOVIE	Delete UR	Delete PD	Delete BCA	Delete CCO	Delete NPTVO	Delete EC	Delete TCC	Delete GCRBD
Nanjing	0.0195	0.0187													
Wuxi	1.0000									0.3156					
Xuzhou	0.7436					0.7186									
Changzhou	0.2706					0.2681								0.2564	
Suzhou	0.2991	0.2983	1.0000							0.2641					0.2886
Nantong	1.0000	0.3030	0.3060	0.3820					0.3226				0.3226		
Lianyungang	1.0000														
Huai’an	1.0000								0.4609		0.5513				
Yancheng	1.0000								0.3488						
Yangzhou	0.3362		0.3361			0.3362			0.3424		0.3362	0.3424	0.3362	0.3217	
Zhenjiang	1.0000														
Taizhou	0.4671		0.4581												
Suqian	1.0000														

**Table A8:** The changes of stochastic efficiency value by deleting grouping input variables in Southern Su (α = 0.95).

Years	DMUs	*a* = 0.95	Delete MF	Delete ID	Delete SP	Delete T	Delete EU	Delete EP
2013	Nanjing	0.6294	0.5882					
	Wuxi	1	0.6848		0.6259			
	Xuzhou	1	0.6755				0.6428	
	Suzhou	0.5162	0.5055		0.5146			0.4946
	Zhenjiang	1			0.6199			
2014	Nanjing	0.6957	0.6098					
	Wuxi	1.0000			0.5222			
	Xuzhou	0.5389			0.5234	0.5361		
	Suzhou	0.5025	0.5047		0.4561			0.4770
	Zhenjiang	1.0000			0.5987			
2015	Nanjing	0.1912	0.1761					
	Wuxi	1.0000			0.4562			
	Xuzhou	1.0000	0.3855					
	Suzhou	0.4761	0.4666		0.4909			0.4849
	Zhenjiang	1.0000			0.3984			
2016	Nanjing	0.0195	0.0187					
	Wuxi	1.0000			0.3156			
	Xuzhou	0.2706	0.2615				0.2499	
	Suzhou	0.2991	0.2833		0.2641			0.2886
	Zhenjiang	1.0000			0.1833			

**Table A9:** The changes of stochastic efficiency value by deleting grouping input variables in Central Su (α = 0.95).

Years	DMUs	*a* = 0.95	Delete MF	Delete ID	Delete SP	Delete T	Delete EU	Delete EP
2013	Nantong	0.5776	0.6129		0.5708	1		
	Yangzhou	0.6181	0.6062		0.6122			
	Taizhou	0.7197	0.6883			0.7265	0.641	0.722
2014	Nantong	1.0000	0.3982			0.4915		
	Yangzhou	0.5401			0.5064			
	Taizhou	0.8035	0.7725			0.7753		0.7675
2015	Nantong	0.5489	0.5007					
	Yangzhou	0.3427	0.3393				0.3151	
	Taizhou	0.5322						0.5251
2016	Nantong	1.0000	0.2984		0.3226		0.3226	
	Yangzhou	0.3362	0.3361		0.3424	0.3362	0.3217	
	Taizhou	0.4671	0.4581					

**Table A10:** The changes of stochastic efficiency value by deleting grouping input variables in Northern Su (α = 0.95).

Years	DMUs	*a* = 0.95	Delete MF	Delete ID	Delete SP	Delete T	Delete EU	Delete EP
2013	Xuzhou	1.0000	0.7798					
	Lianyungang	1.0000						
	Huai’an	1.0000			0.6927	0.8089		
	Yancheng	1.0000			0.6284			
	Suqian	1.0000						
2014	Xuzhou	1.0000	0.6689					
	Lianyungang	1.0000						
	Huai’an	1.0000			0.5820	0.7774		
	Yancheng	1.0000			0.3721			
	Suqian	1.0000						
2015	Xuzhou	0.8004						
	Lianyungang	1.0000			0.4622			
	Huai’an	1.0000			0.4864			
	Yancheng	1.0000			0.3725			
	Suqian	1.0000						
2016	Xuzhou	0.7436	0.7186					
	Lianyungang	1.0000						
	Huai’an	1.0000			0.4609			
	Yancheng	1.0000			0.3222			
	Suqian	1.0000						

**Table A11:** The changes of stochastic efficiency value by deleting grouping input variables in Coastal Area (α = 0.95).

Years	DMUs	*a* = 0.95	Delete MF	Delete ID	Delete SP	Delete T	Delete EU	Delete EP
2013	Nantong	0.5776	0.6129		0.5708	1.0000		
	Lianyungang	1.0000						
	Yancheng	1.0000			0.6284			
2014	Nantong	1.0000	0.3982			0.4915		
	Lianyungang	1.0000						
	Yancheng	1.0000			0.3721			
2015	Nantong	0.5489	0.5007					
	Lianyungang	1.0000			0.4622			
	Yancheng	1.0000			0.3725			
2016	Nantong	1.0000	0.2984		0.3226		0.3226	
	Lianyungang	1.0000						
	Yancheng	1.0000			0.3222			

**Table A12:** The changes of stochastic efficiency value by deleting grouping input variables in Inland Area (α = 0.95).

Years	DMUs	*a* = 0.95	Delete MF	Delete ID	Delete SP	Delete T	Delete EU	Delete EP
2013	Nanjing	0.6294	0.5882					
	Xuzhou	1	0.7798					
	Changzhou	1	0.6755				0.6428	
	Suzhou	0.5162	0.5055		0.5146			0.4946
	Yangzhou	0.6181	0.6062		0.6122			
	Taizhou	0.7197	0.6883			0.7265	0.641	0.722
	Wuxi	1	0.6848		0.6259			
	Zhenjiang	1			0.6199			
	Huai’an	1			0.6927	0.8089		
	Suqian	1						
2014	Nanjing	0.6957	0.6098					
	Xuzhou	1.0000	0.6689					
	Changzhou	0.5389			0.5234	0.5361		
	Suzhou	0.5025	0.5047		0.4561			0.4770
	Yangzhou	0.5401			0.5064			
	Taizhou	0.8035	0.7725			0.7753		0.7675
	Wuxi	1.0000			0.5222			
	Zhenjiang	1.0000			0.5987			
	Huai’an	1.0000			0.5820	0.7774		
	Suqian	1.0000						
2015	Nanjing	0.1912	0.1761					
	Xuzhou	0.8004						
	Changzhou	1.0000	0.3855					
	Suzhou	0.4761	0.4666		0.4909			0.4849
	Yangzhou	0.3427	0.3393				0.3151	
	Taizhou	0.5322						0.5251
	Wuxi	1.0000			0.4562			
	Zhenjiang	1.0000			0.3984			
	Huai’an	1.0000			0.4864			
	Suqian	1.0000						
2016	Nanjing	0.0195	0.0187					
	Xuzhou	0.7436	0.7186					
	Changzhou	0.2706	0.2615				0.2499	
	Suzhou	0.2991	0.2833		0.2641			0.2886
	Yangzhou	0.3362	0.3361		0.3424	0.3362	0.3217	
	Taizhou	0.4671	0.4581					
	Wuxi	1.0000			0.3156			
	Zhenjiang	1.0000			0.1833			
	Huai’an	1.0000			0.4609			
	Suqian	1.0000						

**Table A13:** The changes of stochastic efficiency value by deleting single input variable in Southern Su (α = 0.95).

Years	DMUs	*a* = 0.95	Delete WS	Delete NPD	Delete PTC	Delete NPC	Delete RH	Delete GOVIE	Delete UR	Delete PD	Delete BCA	Delete CCO	Delete NPTVO	Delete EC	Delete TCC	Delete GCRBD
2013	Nanjing	0.6294	0.5834				1.0000									
	Wuxi	1.0000					0.6760				0.6259					
	Xuzhou	1.0000	0.6866				0.6755								0.6805	
	Suzhou	0.5162					0.5055			0.5146						0.4946
	Zhenjiang	1.0000									0.6199					
2014	Nanjing	0.6957	0.6494				0.6098									
	Wuxi	1.0000									0.5222					
	Xuzhou	0.5389									0.5234		0.5361			
	Suzhou	0.5025	0.5047								0.4561					0.4770
	Zhenjiang	1.0000									0.5987					
2015	Nanjing	0.1912	0.1761													
	Wuxi	1.0000									0.4562					
	Xuzhou	1.0000					0.3920									
	Suzhou	0.4761	0.4618	0.4842			0.4761			0.4757	0.4909					0.4849
	Zhenjiang	1.0000									0.3984					
2016	Nanjing	0.0195	0.0187													
	Wuxi	1.0000									0.3156					
	Xuzhou	0.2706					0.2681								0.2564	
	Suzhou	0.2991	0.2983	1.0000							0.2641					0.2886
	Zhenjiang	1.0000														

**Table A14:** The changes of stochastic efficiency value by deleting single input variable in Central Su (α = 0.95).

Years	DMUs	*a* = 0.95	Delete WS	Delete NPD	Delete PTC	Delete NPC	Delete RH	Delete GOVIE	Delete UR	Delete PD	Delete BCA	Delete CCO	Delete NPTVO	Delete EC	Delete TCC	Delete GCRBD
2013	Nantong	0.5776	0.6129						0.5708				1.0000			
	Yangzhou	0.6181					0.6062			0.6122						
	Taizhou	0.7197	0.6883										0.7265	0.6410		0.7220
2014	Nantong	1.0000	0.3982										0.4915			
	Yangzhou	0.5401								0.5064						
	Taizhou	0.8035	0.7725										0.7837			0.7675
2015	Nantong	0.5489	0.5221	0.5364												
	Yangzhou	0.3427		0.3393											0.3151	
	Taizhou	0.5322														0.5251
2016	Nantong	1.0000	0.3030	0.3060	0.3820					0.3226				0.3226		
	Yangzhou	0.3362		0.3361			0.3362			0.3424		0.3362	0.3424	0.3362	0.3217	
	Taizhou	0.4671		0.4581												

**Table A15:** The changes of stochastic efficiency value by deleting single input variable in Northern Su (α = 0.95).

Years	DMUs	*a* = 0.95	Delete WS	Delete NPD	Delete PTC	Delete NPC	Delete RH	Delete GOVIE	Delete UR	Delete PD	Delete BCA	Delete CCO	Delete NPTVO	Delete EC	Delete TCC	Delete GCRBD
2013	Xuzhou	1.0000					0.8123									
	Lianyungang	1.0000														
	Huai’an	1.0000								0.7173		0.8089				
	Yancheng	1.0000														
	Suqian	1.0000														
2014	Xuzhou	1.0000					0.6790									
	Lianyungang	1.0000														
	Huai’an	1.0000								0.6079		0.7774				
	Yancheng	1.0000								0.4315						
	Suqian	1.0000														
2015	Xuzhou	0.8004														
	Lianyungang	1.0000									0.4622					
	Huai’an	1.0000								0.4883						
	Yancheng	1.0000								0.4026						
	Suqian	1.0000														
2016	Xuzhou	0.7436					0.7186									
	Lianyungang	1.0000														
	Huai’an	1.0000								0.4609		0.5513				
	Yancheng	1.0000								0.3488						
	Suqian	1.0000														

**Table A16:** The changes of stochastic efficiency value by deleting single input variable in Coastal Area (α = 0.95).

Years	DMUs	*a* = 0.95	Delete WS	Delete NPD	Delete PTC	Delete NPC	Delete RH	Delete GOVIE	Delete UR	Delete PD	Delete BCA	Delete CCO	Delete NPTVO	Delete EC	Delete TCC	Delete GCRBD
2013	Nantong	0.5776	0.6129						0.5708				1.0000			
	Lianyungang	1.0000														
	Yancheng	1.0000														
2014	Nantong	1.0000	0.3982										0.4915			
	Lianyungang	1.0000														
	Yancheng	1.0000								0.4315						
2015	Nantong	0.5489	0.5221	0.5364												
	Lianyungang	1.0000									0.4622					
	Yancheng	1.0000								0.4026						
2016	Nantong	1.0000	0.3030	0.3060	0.3820					0.3226				0.3226		
	Lianyungang	1.0000														
	Yancheng	1.0000								0.3488						

**Table A17:** The changes of stochastic efficiency value by deleting single input variable in Inland Area (α = 0.95).

Years	DMUs	*a* = 0.95	Delete WS	Delete NPD	Delete PTC	Delete NPC	Delete RH	Delete GOVIE	Delete UR	Delete PD	Delete BCA	Delete CCO	Delete NPTVO	Delete EC	Delete TCC	Delete GCRBD
2013	Nanjing	0.6294	0.5834				1.0000									
	Xuzhou	1.0000					0.8123									
	Changzhou	1.0000	0.6866				0.6755								0.6805	
	Suzhou	0.5162					0.5055			0.5146						0.4946
	Yangzhou	0.6181					0.6062			0.6122						
	Taizhou	0.7197	0.6883										0.7265	0.6410		0.7220
	Wuxi	1.0000					0.6760				0.6259					
	Zhenjiang	1.0000									0.6199					
	Huai’an	1.0000								0.7173		0.8089				
	Suqian	1.0000														
2014	Nanjing	0.6957	0.6494				0.6098									
	Xuzhou	1.0000					0.6790									
	Changzhou	0.5389									0.5234		0.5361			
	Suzhou	0.5025	0.5047								0.4561					0.4770
	Yangzhou	0.5401								0.5064						
	Taizhou	0.8035	0.7725										0.7837			0.7675
	Wuxi	1.0000									0.5222					
	Zhenjiang	1.0000									0.5987					
	Huai’an	1.0000								0.6079		0.7774				
	Suqian	1.0000														
2015	Nanjing	0.1912	0.1761													
	Xuzhou	0.8004														
	Changzhou	1.0000					0.3920									
	Suzhou	0.4761	0.4618	0.4842			0.4761			0.4757	0.4909					0.4849
	Yangzhou	0.3427		0.3393											0.3151	
	Taizhou	0.5322														0.5251
	Wuxi	1.0000									0.4562					
	Zhenjiang	1.0000									0.3984					
	Huai’an	1.0000								0.4883						
	Suqian	1.0000														
2016	Nanjing	0.0195	0.0187													
	Xuzhou	0.7436					0.7186									
	Changzhou	0.2706					0.2681								0.2564	
	Suzhou	0.2991	0.2983	1.0000							0.2641					0.2886
	Yangzhou	0.3362		0.3361			0.3362			0.3424		0.3362	0.3424	0.3362	0.3217	
	Taizhou	0.4671		0.4581												
	Wuxi	1.0000									0.3156					
	Zhenjiang	1.0000														
	Huai’an	1.0000								0.4609		0.5513				
	Suqian	1.0000														
